# The Nitric Oxide (NO) Donor Molsidomine Attenuates Memory Impairments Induced by the D1/D2 Dopaminergic Receptor Agonist Apomorphine in the Rat

**DOI:** 10.3390/molecules28196861

**Published:** 2023-09-28

**Authors:** Foteini Vartzoka, Elif Ozenoglu, Nikolaos Pitsikas

**Affiliations:** 1 Department of Pharmacology, Faculty of Medicine, School of Health Sciences, University of Thessaly, Biopolis, Panepistimiou 3, 415-00 Larissa, Greece; 2 School of Medicine, University of Acibadem, 415-00 Istanbul, Turkey

**Keywords:** nitric oxide, dopamine, schizophrenia, molsidomine, memory, rat

## Abstract

Several lines of evidence suggest that scarcity of the gaseous molecule nitric oxide (NO) is associated with the pathogenesis of schizophrenia. Therefore, compounds, such as NO donors, that can normalize NO levels might be of utility for the treatment of this pathology. It has been previously shown that the NO donor molsidomine attenuated schizophrenia-like behavioral deficits caused by glutamate hypofunction in rats. The aim of the current study was to investigate the efficacy of molsidomine and that of the joint administration of this NO donor with sub-effective doses of the non-typical antipsychotics clozapine and risperidone to counteract memory deficits associated with dysregulation of the brain dopaminergic system in rats. Molsidomine (2 and 4 mg/kg) attenuated spatial recognition and emotional memory deficits induced by the mixed dopamine (DA) D_1_/D_2_ receptor agonist apomorphine (0.5 mg/kg). Further, the joint administration of sub-effective doses of molsidomine (1 mg/kg) with those of clozapine (0.1 mg/kg) or risperidone (0.03 mg/kg) counteracted non-spatial recognition memory impairments caused by apomorphine. The present findings propose that molsidomine is sensitive to DA dysregulation since it attenuates memory deficits induced by apomorphine. Further, the current findings reinforce the potential of molsidomine as a complementary molecule for the treatment of schizophrenia.

## 1. Introduction

Schizophrenia is a severe, chronic psychiatric disorder. Up to 1% of the world’s inhabitants seem to be afflicted by schizophrenia. This disease is usually revealed in late youth or early maturity. Quality of life is critically impaired in schizophrenia [1]. Schizophrenia symptoms can be divided into three categories: positive symptoms (hallucinations, delusions, thought disturbances, hypermotility, stereotypies, and confused speech); negative symptoms (flat expression, anhedonia, alogia, avolition, and social withdrawal); and cognition deficits (disturbances of attention, executive functioning, and memory) [2].

The outcome of a plethora of studies proposes that the functionality of the dopaminergic (DAergic) system is seriously compromised in schizophrenia. It has been shown that cognitive deficits are associated with reduced prefrontal cortex (PFC) DAergic activity [3]. Other reports have demonstrated that either scarce or exaggerated DA stimulation disrupts cognitive performance either in animals or in humans [4,5,6,7].

Currently available neuroleptics, including clozapine and risperidone, can only control positive symptoms. These medications are unable to provide relief for the negative symptoms and cognitive disturbances associated with schizophrenia and are also correlated with serious side effects. Treatment with clozapine can cause agranulocytosis and low neutrophil levels, while treatment with risperidone is associated with weight gain, hyperlipidemia, and tachycardia. Further, 30% of patients are insensitive to any pharmacological intervention. This suggests a pressing need for new medications with superior efficacy and safety with respect to the traditional antipsychotics, which might be able to alleviate negative symptoms and reduce cognitive deficits in all patients [8].

Nitric oxide (NO) is a soluble, unstable, highly diffusible, and short-lived gas. NO is considered to be an intra- and inter-cellular messenger in the brain [9]. In a series of studies, the involvement of NO in schizophrenia has been evidenced (for review, please see [10]). In particular, it has been proposed that underproduction of NO appears to be correlated with schizophrenia [10]. Therefore, regularization of the brain concentrations of NO in schizophrenia patients could be of therapeutic utility in this context. In agreement with the above, compounds that can increase NO production, such as the NO donors, could serve as a new therapeutic approach targeting the alleviation of psychotic symptoms. This perspective has been supported by preclinical results. Specifically, the ability of various NO donors to alleviate psychotomimetic effects and memory impairments in different pharmacological animal models of schizophrenia has been observed (for review, please see [11]).

By contrast, the promising preclinical results did not find replication in clinical studies. The effectiveness of the NO donor sodium nitroprusside (SNP) is limited since it is revealed solely in specific groups of schizophrenics (young, no smokers, and in the early phase of the disease) [11,12]. Additionally, challenges with SNP are frequently accompanied by undesirable side effects such as methemoglobinemia [13] and sedation [14]. Further, SNPs pharmacological activity is denoted by a limited therapeutic window [15].

Molsidomine is a NO donor and, like SNP, is utilized for the therapy of angina pectoris. Molsidomine is a prodrug and is rapidly metabolized into its active metabolite, 3-morpholinosydnonimine (SIN-1). Molsidomine mediates its effects through NO, increases myocardial perfusion by vasodilating the coronary artery system, and reduces oxygen demand by increasing peripheral venous capacitance, cardiac preload, and wall tension [16]. Importantly, molsidomine does not produce methemoglobinemia and has a longer duration of action (2 h) [17] compared to that expressed by SNP (4 min) [18]. Molsidomine was shown to attenuate psychotomimetic effects, social isolation, and recognition memory deficits caused by treatment with the N-methyl-D-aspartate (NMDA) receptor antagonists MK-801 and ketamine [19,20]. Further, co-administration of inactive doses of molsidomine and the atypical neuroleptic clozapine counteracted the recognition memory produced by ketamine [20].

Presently, little information is available concerning the ability of molsidomine to attenuate cognitive impairments caused by dysfunction of the DAergic system. In this context, a preliminary study reported that molsidomine reduced non-spatial recognition memory deficits induced by the mixed D_1_/D_2_ DA receptor agonist apomorphine [7]. It is still not elucidated, however, whether molsidomine can counteract spatial recognition and emotional memory deficits produced by acute challenges with apomorphine. It is well documented that apomorphine causes schizophrenia-like symptoms, comprising cognitive impairments, either in humans or animals [7,21,22,23]. It is important to underline that both recognition and emotional memory are cognitive domains severely impaired in schizophrenia patients [24,25].

Hence, the first aim of the present study was to test in rats the ability of molsidomine to attenuate spatial recognition and emotional memory impairments produced by apomorphine. For these experiments, the object location task (OLT), a behavioral test assessing spatial recognition memory [26], and the step-through passive avoidance test (STPAT), a behavioral paradigm assessing emotional memory [27], were used, respectively. Afterwards, we aimed to test whether the concomitant administration of inactive doses of molsidomine with inactive doses of the second-generation antipsychotics clozapine and risperidone could attenuate delay-dependent and apomorphine-induced non-spatial recognition memory impairments. A potential utility of the jointed treatment schedule is that the reduction of compound doses might rule out the serious adverse effects of them described above. Accordingly, it has been previously demonstrated that the combination of inactive doses of SNP with clozapine reversed attentional deficits caused by the DA releaser amphetamine in mice [28]. For this second set of experiments, the object recognition task (ORT), a procedure examining non-spatial recognition memory in rodents, was used [29].

## 2. Results

### 2.1. Experiment 1: Effects of Acute Treatment with Molsidomine on Attenuating Apomorphine-Induced Performance Deficits in the OLT

**Figure 1:**
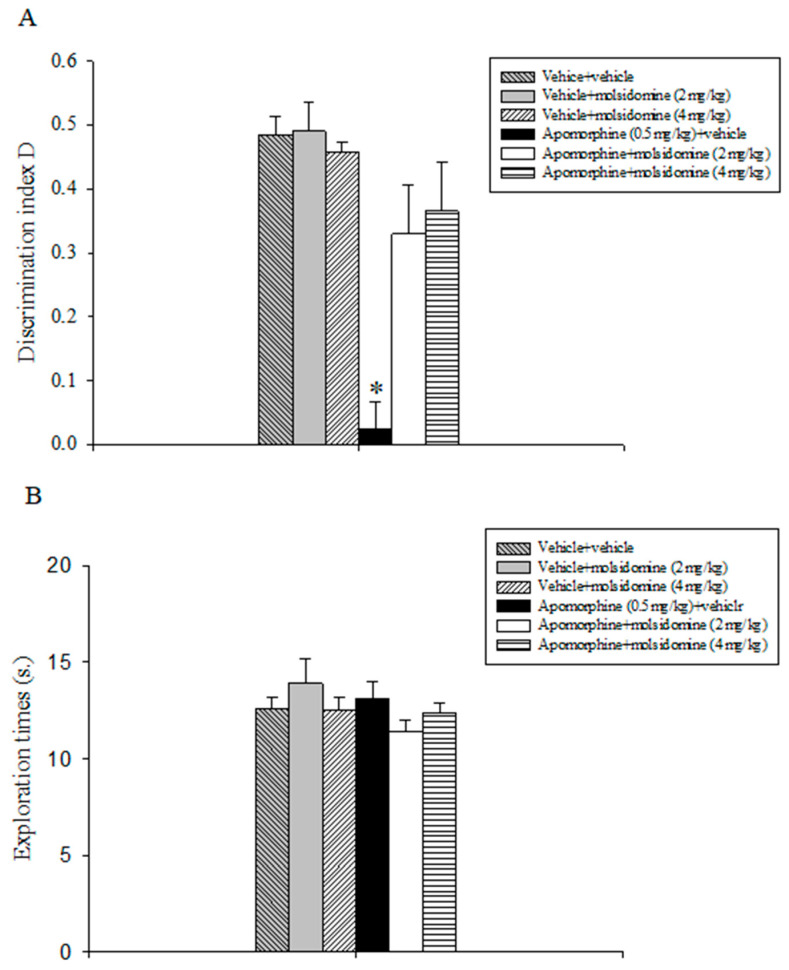
Object location task. The histogram represents the mean ± S.E.M. of 8 rats per treatment group. (**A**) Discrimination index D performance expressed by various groups of rats during the choice phase (T2). * *p* < 0.05 vs. all the other groups. (**B**) Total exploration times.

Results are illustrated in Figure 1. Statistical analysis of the D index data evidenced a statistically significant main effect of apomorphine (F(1,47) = 25.149, *p* < 0.001), of molsidomine (F(2,47) = 5.991, *p* = 0.005), and a significant apomorphine x molsidomine interaction (F(2,47) = 6.769, *p* = 0.003). Post-hoc analysis revealed that the animals treated with apomorphine and vehicle were unable to discriminate between the novel and the familiar object as to all the other treatment groups, including the apomorphine + 2 mg/kg molsidomine and apomorphine + 4 mg/kg molsidomine groups (*p* < 0.05; Figure 1A).

The evaluation of the exploratory activity data expressed by the various rats’ populations during the choice trial T2 did not show statistically significant effects of apomorphine, molsidomine, or their combination (Figure 1B).

### 2.2. Experiment 2: Effects of Acute Treatment with Molsidomine on Attenuating Apomorphine-Induced Performance Deficits in the STPAT

Pre-shock latencies did not vary among the different treatment groups; H(5) = 0.586; *p* = 0.989. By contrast, the Kruskal–Wallis test evidenced statistically significant differences regarding the retention post-shock latencies (H(5) = 19.157, *p* = 0.002). Post-hoc tests conducted on the data showed that the apomorphine + vehicle group expressed poor retention latencies compared to all the other treatment groups, including apomorphine + 2 mg/kg molsidomine and apomorphine + 4 mg/kg molsidomine (*p* < 0.05) (Table 1).

### 2.3. Experiment 3: Effects of Acute Treatment with Sub-Threshold Doses of Molsidomine and Clozapine on Attenuating Apomorphine-Induced Performance Deficits in the ORT

**Figure 2:**
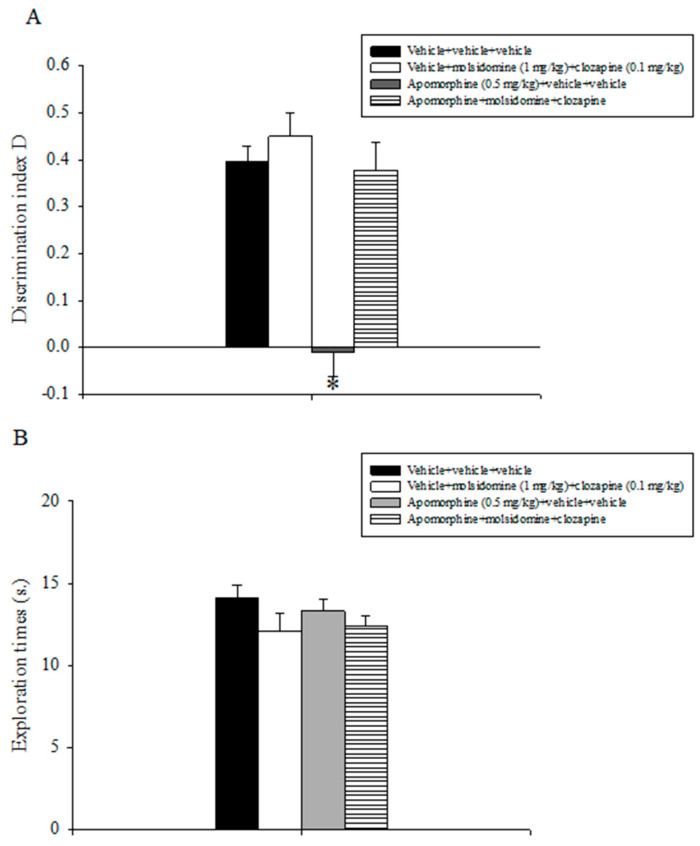
Object recognition task. The histogram represents the mean ± S.E.M. of 8 rats per treatment group. (**A**) Discrimination index D performance expressed by various groups of rats during the choice phase (T2). * *p* < 0.05 vs. all the other groups. (**B**) Total exploration times.

Relative results are reported in Figure 2. The analysis of the discrimination index D data revealed a statistically significant main effect of apomorphine (F(1,31) = 35.071, *p* < 0.001), of the combination between molsidomine and clozapine (F(1,31) = 30.881, *p* < 0.001), and a significant interaction between apomorphine and the combination of inactive doses of molsidomine and clozapine (F(1,31) = 6.544, *p* = 0.016). Post-hoc comparisons evidenced that the apomorphine + vehicle +vehicle group was unable to discriminate between the novel and the familiar object as compared to all the other experimental groups, including the apomorphine + molsidomine + clozapine group (*p* < 0.05; Figure 2A).

Analysis of the exploratory activity data displayed by the various groups of animals during T2 did not evidence statistically significant effects of apomorphine, molsidomine, clozapine, or their combination (Figure 2B).

### 2.4. Experiment 4: Effects of Acute Treatment with Sub-Threshold Doses of Molsidomine and Risperidone on Natural Forgetting Assessed in the ORT

**Figure 3:**
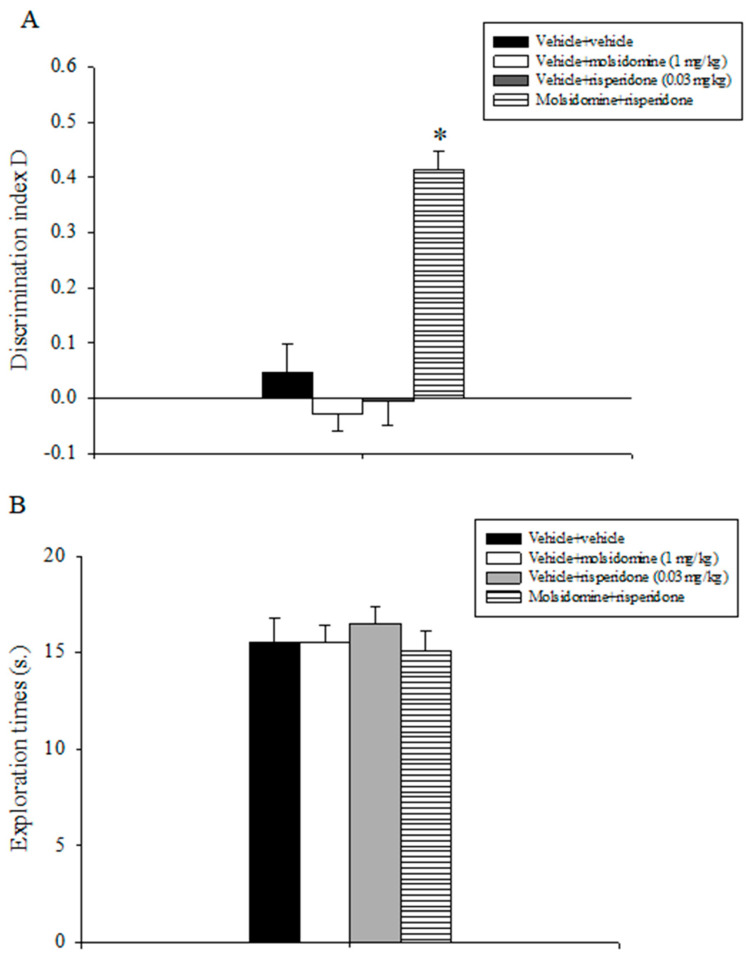
Object recognition task. The histogram represents the mean ± S.E.M. of 8 rats per treatment group. (**A**) Discrimination index D performance expressed by various groups of rats during the choice phase (T2). * *p* < 0.05 vs. all the other groups. (**B**) Total exploration times.

Statistical analyses conducted on the discrimination index D data revealed a statistically significant main effect of molsidomine (F(1,31) = 21.813, *p* < 0.001), of risperidone (F(1,31) = 18.109, *p* < 0.001), and a significant molsidomine x risperidone interaction (F(1,31) = 34.955, *p* < 0.001). Post-hoc comparisons conducted on the data revealed that the animals that received the combination treatment of the inactive doses of molsidomine and risperidone expressed a higher discrimination index D as compared to all the other experimental groups (*p* < 0.05; Figure 3A).

The overall assessment of the total exploration times expressed by the various groups of animals during T2 did not evidence any statistically significant effect of treatments (Figure 3B).

### 2.5. Experiment 5: Effects of Acute Treatment with Sub-Threshold Doses of Molsidomine and Risperidone on Attenuating Apomorphine-Induced Performance Deficits in the ORT

**Figure 4:**
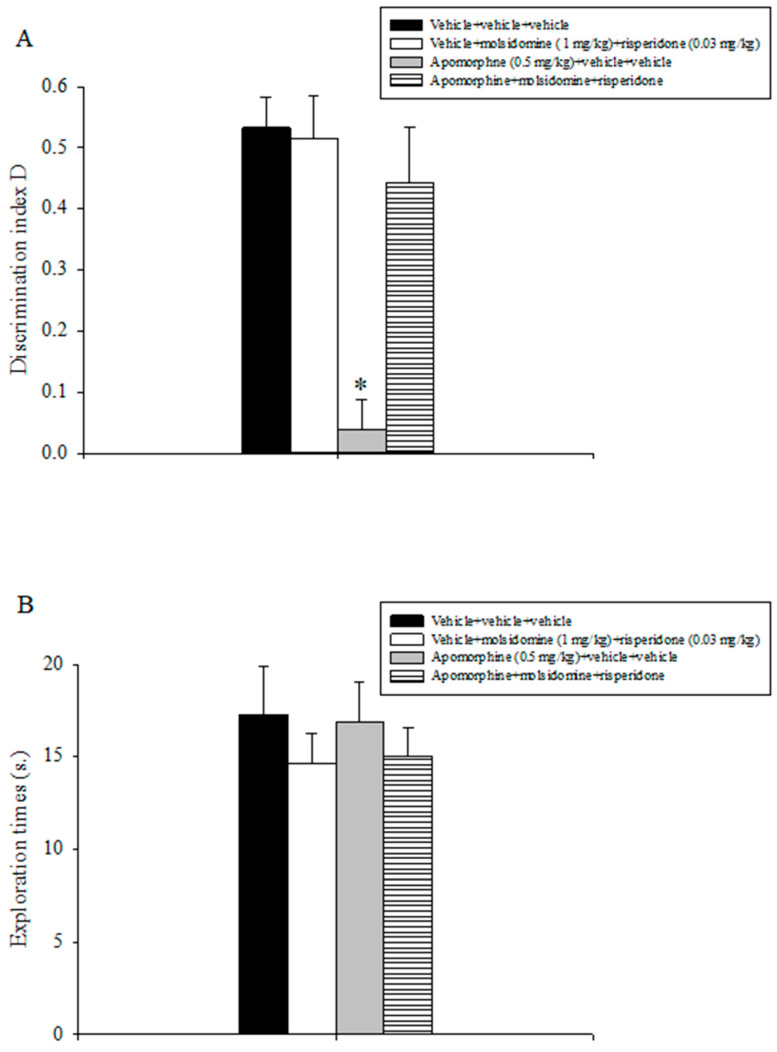
Object recognition task. The histogram represents the mean ± S.E.M. of 8 rats per treatment group. (**A**) Discrimination index D performance expressed by various groups of rats during the choice phase (T2). * *p* < 0.05 vs. all the other groups. (**B**) Total exploration times.

Results are outlined in Figure 4. The evaluation of the discrimination index D data showed a statistically significant main effect of apomorphine (F(1,31) = 18.481, *p* < 0.001), of the combination between molsidomine and risperidone (F(1,31) = 8.608, *p* = 0.007), and a significant interaction between apomorphine and the combination of sub-threshold doses of molsidomine and risperidone (F(1,31) = 10.244, *p* = 0.003). Post-hoc comparisons indicated that the apomorphine + vehicle + vehicle group expressed a lower discrimination index D as compared to all the other treatment groups, including the apomorphine + molsidomine + clozapine group (*p* < 0.05; Figure 4A).

Statistical analysis conducted on the exploratory activity data displayed by the various populations of animals during T2 did not detect statistically appreciable effects of apomorphine, molsidomine, risperidone, or their combination (Figure 4B).

## 3. Discussion

In line with prior results, post-training acute challenges with apomorphine disrupted spatial recognition memory in rats, as evidenced in the OLT [30]. Molsidomine, at any dose tested (2–4 mg/kg), attenuated apomorphine’s inhibitory effect on rats’ spatial recognition memory abilities. These results extend antecedent findings in which this NO donor counteracted disruption of spatial recognition memory caused by blockade of the NMDA receptor [20] and impairments of non-spatial recognition memory induced by apomorphine [7]. The present findings are also in agreement with previous work in which SNP counteracted attentional deficits induced by the DA releaser amphetamine in mice [31].

In line with previous findings, apomorphine (0.5 mg/kg) compromised rats’ performance in the STPAT, indicating a deleterious effect of it on emotional memory [23,30]. Acute administration of molsidomine attenuated this impairing effect of apomorphine on emotional memory. Either in the OLT or in the STPAT, molsidomine alone did not affect animals’ cognitive performance.

In a subsequent study, the efficacy of the joint treatment of inactive doses of molsidomine with those of the atypical neuroleptic risperidone to reduce delay-dependent non-spatial recognition memory impairments was examined in the ORT. Post-training challenge with this combination significantly reduced delay-dependent non-spatial recognition memory deficits. By contrast, the sub-effective doses of molsidomine and risperidone by themselves did not improve the time-dependent extinction of recognition memory.

Finally, the effectiveness of the concomitant administration of sub-threshold doses of molsidomine with sub-threshold doses either of clozapine or risperidone to counteract non-spatial recognition memory deficits caused by apomorphine was assessed using the ORT. The joint administration of sub-effective doses of molsidomine and clozapine or risperidone alleviated the disrupting effects of apomorphine on rodents’ non-spatial recognition memory abilities. A per se effect of the combination treatment between molsidomine and clozapine or risperidone on recognition memory was not observed.

Thus far, few studies have examined the prospective anti-schizophrenia-like effects of the concomitant administration of a NO donor with a currently used antipsychotic (i.e., olanzapine, clozapine, or risperidone). Previous research has demonstrated that co-administration of SNP with olanzapine or risperidone improved rats’ performance in the conditioned avoidance response test (CART), a paradigm mimicking the positive symptoms of schizophrenia [32,33]. In line with the above, we have previously shown that joint administration of inactive doses of molsidomine with clozapine attenuated non-spatial recognition memory caused by blockade of the NMDA receptor in rats [20]. The results here reported indicate that a combination of sub-threshold doses of molsidomine, either with sub-threshold doses of clozapine or risperidone, alleviated non-spatial recognition memory impairments caused by DAergic dysfunction. These findings reply to and extend the results of a prior report in which concomitant administration of SNP with clozapine attenuated attentional deficits in mice caused by amphetamine [28].

The present findings suggest a synergistic action of the combined treatment with relevance to recognition memory, a cognitive domain impaired in schizophrenia patients. Further, jointly with previous findings [20], the current results propose to investigate the clinical usefulness of molsidomine as a supplementary therapy to antipsychotic drugs in schizophrenia.

OLT and ORT are two behavioral paradigms assessing spatial and non-spatial recognition memory in rodents [26,29]. Interestingly, these paradigms do not imply explicit reward or punishment but are dependent on the innate inquisitiveness of rodents, are almost identical to procedures utilized in clinical trials, and thus display congruous predictive validity [29]. According to the experimental protocols, the effects displayed by the various chemicals either in the OLT or ORT could suggest a possible regulation of post-training memory components (consolidation and/or retrieval of information). In the studies in which a short intertrial interval (ITI) between the training and the retention trial (2 h) was posed, it is hard to distinguish the effects of compounds on consolidation from potential effects on retrieval. Additionally, due to the short ITI applied, the paradigms utilized can be intended as short-term memory tests.

Conversely, in the ORT study, in which the effects of the concomitant administration of the inactive doses of molsidomine with those of risperidone were assessed on natural forgetting, a long ITI (24 h) was applied. As the relative half-lives of molsidomine, clozapine, and risperidone are 2, 6, and 2.5 h, respectively [17,34,35], it might be assumed that the effects of molecules were revealed in a procedure testing long-term memory. Accordingly, this joint treatment appears to act expressly on the consolidation memory stage.

In all recognition memory studies, compounds were delivered intraperitoneally. It cannot be ruled out, thus, that unspecific components (e.g., sensorimotor or motivational) could have influenced animals’ mnemonic outcomes. Since the exploration levels expressed by rats during the choice trial T2 in all recognition memory tasks were superimposable among the various treatment groups, it can be concluded that nonspecific factors did not affect rats’ cognitive performance.

Drugs injected before the animals’ exposure to the noxious stimulus in the STPAT might influence different parameters unrelated to cognition (e.g., motility, pain perception). On this account, it is important to emphasize that pre-shock latencies did not vary among the different experimental groups, and the dose of apomorphine (0.5 mg/kg) used did not affect pain perception in rats [23]. Further, molsidomine was found to induce an analgesic effect in mice at a dose range (150–300 mg/kg) consistently higher than that (2–4 mg/kg) used in the present study [36]. Overall, these findings indicate that the involvement of ambiguous factors in the effects of compounds on animals’ cognitive performance in the STPAT might probably be excluded.

The mechanism(s) underlying apomorphine’s deleterious effects on cognition have been ascribed, at least, to the activation of the D_2_ DA receptor on PFC [37]. It has also been reported that apomorphine exerts an inhibitory effect on long-term potentiation (LTP), which is considered an electrophysiological correlate of learning [38].

The mechanism(s) of action by which molsidomine attenuates apomorphine’s detrimental action on memory is still a matter of investigation. In this context, it has been shown that different NO donors, including molsidomine, promote LTP formation [39,40]. Further, the involvement of the nucleotide cAMP in that respect might be critical. Specifically, it has been reported that the attentional deficits induced by apomorphine and amphetamine in mice are accompanied by an abnormal increase of the nucleotide cAMP. SNP counteracted the above-mentioned attentional deficits and normalized cAMP levels [28]. It has also been proposed that NO donors might attenuate the amnestic action of apomorphine by inhibiting its stimulatory action on the D_2_ DA receptor [7].

An alternative hypothesis that might justify the results here presented is based on the well-known association between schizophrenia and oxidative stress [41]. In a series of studies, the pro-oxidant profile of apomorphine has been evidenced [38,42,43]. Thus, the anti-oxidant profile of NO donors, including molsidomine, as evidenced in a vast number of experimental models (for review, please see [44]), may provide a plausible explanation of molsidomine’s beneficial effects.

The potential mechanism(s) of action underlying the beneficial effects of the joint treatment on recognition memory is not yet clarified. Within this framework, it has been shown that both clozapine and risperidone enhance DA production in the medial PFC (mPFC) and hippocampus in rats [45,46]. It seems that the enhancing action of atypical antipsychotics might be critical for their beneficial effects on cognitive disturbances revealed in schizophrenia [47]. In support of the above, it has been reported that SNP reinforced risperidone’s anti-schizophrenia-like effect by stimulating the risperidone-mediated outflow of DA in the mPFC, a brain structure that plays an important role in cognition [31]. The latter might represent a potential hypothesis to explain the effectiveness of the joint treatment on recognition and memory impairments.

The stimulatory action exerted by molsidomine and atypical neuroleptics on cholinergic transmission might be an alternative explanation for the concomitant treatment results. It has been demonstrated that molsidomine [48], clozapine [46,49], and risperidone [50] stimulate the release of acetylcholine (Ach), a neurotransmitter consistently implicated in cognition, in different brain areas. In addition, the antioxidant properties of molsidomine, clozapine, and risperidone [44,51] might also play a role in this context. Molsidomine [40], clozapine [52], but not risperidone [52], which were found efficacious to potentiate synaptic efficacy by inducing LTP, might also be inspected for the evaluation of the joint treatment findings.

The current study has some limitations. The results here presented might be considered preliminary and are limited to behavioral findings. Additional intense research (e.g., molecular, biochemical, and electrophysiological studies) is required to fully elucidate the mechanism(s) of action by which either molsidomine’s or the jointed treatment produced their anti-amnestic effects.

## 4. Materials and Methods

### 4.1. Animals

Different populations of male (3-month-old) Wistar rats (Hellenic Pasteur Institute, Athens, Greece) weighing 250–300 g were used for each study. The animals were housed in Makrolon cages (47.5 cm length × 20.5 cm height × 27 cm width), with three per cage, in a standard environment (21 ± 1 °C; 50–55% relative humidity; 12 h/12 h light/dark cycle, lights on at 7 a.m.) with free access to food and water.

The experiments that involved animals and their care were conducted in accordance with international guidelines and national (Animal Act, P.D. 160/91) and international laws and policies (EEC Council Directive 86/609, JL 358, 1, 12 December 1987). The present study was approved by the local committee (Prefecture of Larissa, Greece, protocol number 58379/13 February 2023).

### 4.2. Behavior

#### 4.2.1. Object Location Task (OLT)

The OLT is a behavioral paradigm evaluating spatial recognition memory. This task examines the ability of rodents to discriminate between the novelty of the object locations but not the objects themselves because the testing arena is already familiar to the animals [26]. The test apparatus consisted of a dark open box made of Plexiglas (80 cm length × 50 cm height × 60 cm width) that was illuminated by a 60 W light suspended 60 cm above the box. The light intensity was equal in the different parts of the apparatus. The apparatus was placed in a large observation room and was surrounded with large and typical external objects (cues) to assist the animals in correctly carrying out the test. These cues were kept in a fixed position for the entire testing period. The objects were made of glass, plastic, or metal and were in three different shapes—metallic cubes; glass pyramids; and plastic cylinders 7 cm high—and could not be displaced by rats.

OLT was performed as described elsewhere [20,26]. In brief, during the week before undertaking testing, the rats were handled twice daily for three consecutive days. Before testing, the rats were allowed to explore the empty apparatus for 2 min for 3 consecutive days. During testing, a session that consisted of two 2 min trials was conducted. During the “sample” trial T1, two identical samples (objects) were positioned in two opposite corners of the same side of the apparatus in a casual fashion, 10 cm away from the sidewalls. A rat was gently positioned in the center of the open box and allowed to explore the two similar objects. After the sample phase T1, the rat returned to its home cage, and an intertrial interval (ITI) followed. Afterwards, the “choice” trial T2 was performed. During T2, one of the two similar objects was moved to a different location (new location) (NL), while the other object remained in the same position (FL) as in T1. Therefore, the two objects were now in diagonal corners.

All the combinations and locations of the objects were counterbalanced to eliminate any possible bias due to preferences for particular locations or objects.

Exploration was defined as follows: directing the nose toward the object at a distance of 2 cm or less and/or touching the object with the nose. Turning around or sitting on the object was not considered exploratory behavior. The time spent by the rats exploring each object during T1 and T2 was manually recorded with a stopwatch. The discrimination between the FL and NL during T2 was measured by comparing the time spent exploring the object in the FL with the time spent exploring the object in the NL. Because the exploratory time may be influenced by differences in the total exploratory activity, a discrimination index (D) representing the preference for the new as opposed to familiar object position was calculated as follows: D = NL − FL/NL + FL, whereas NL is the exploration time of the object in the novel location, FL is that of the object in the familiar location, and NL + FL is the total exploration time of both objects during T2 [53]. Correct recognition is shown by rats spending consistently more time inspecting the novel location of the object than the familiar one during choice trial T2 [26].

#### 4.2.2. Step-Through Passive Avoidance Task (STPAT)

STPAT is a one-trial emotional memory test [27]. The test apparatus was composed of a large box with a grid floor (50 cm length × 50 cm height × 50 cm width) made of Plexiglas connected to a platform illuminated by a 60 W lamp suspended 20 cm above the box. The platform was separated from the compartment by a guillotine door. Electric shocks were delivered to the grid floor by an isolated generator.

The procedure described by King and Glasser (1970) [54] was utilized. During the first day (training session), each rat was gently posed on the illuminated platform, and 10 s. later, the guillotine door was opened. As soon as the animal has moved into the dark compartment and the door has been shut, a 0.6 mA foot shock is applied for 1 s [23]. The time spent by each rat to enter the dark chamber was recorded and was considered pre-shock latency. Subsequently, the rat was immediately removed from the apparatus and returned to its home cage. A retention trial was performed 24 h later. Each animal was placed on the illuminated platform, and the step-through latency was recorded. This is the time the rat remained on the illuminated platform. The test was stopped as soon as the animal entered the dark chamber or remained on the lighted platform for 180 s.

#### 4.2.3. Object Recognition Task (ORT)

ORT evaluates non-spatial recognition memory abilities in rodents [29]. The test apparatus was in the same chamber as that utilized in the OLT. The objects to be discriminated against (in triplicate) were the same objects as in the OLT.

ORT was conducted as described previously [20]. Briefly, during the week before undertaking the testing, the rats were handled twice a day for three consecutive days. Before testing, the rats were allowed to explore the empty apparatus for 2 min for 3 consecutive days. During testing, a session that consisted of two 2 min trials was conducted. During the “sample” trial (T1), two identical samples (objects) were positioned in two opposite corners of the apparatus in a casual fashion, 10 cm away from the sidewalls. A rat was gently positioned in the center of the testing box and allowed to explore two similar objects. After the sample phase (T1), the rat returned to its home cage, and an ITI followed. Afterwards, the “choice” trial (T2) was conducted. During T2, a novel object substituted one of the objects presented during T1. The animals, thus, were re-exposed to two objects: a copy of the familiar F object and the novel N object.

All the combinations and positions of the objects were counterbalanced to diminish the possible bias due to preferences for locations or objects. The definition of exploration is provided above in the context of describing the object location procedure.

The total time spent by the rats exploring the two identical objects F1 and F2 during the sample phase T1 and the total time spent exploring the two different objects (F and N) during the choice trial T2 were manually recorded by using a stopwatch. The discrimination between F and N during T2 was measured by comparing the time spent exploring the familiar object with the time spent exploring the novel object. Because the exploratory time may be influenced by differences in the total exploratory activity, a discrimination index D representing the preference for the new as opposed to the familiar object was calculated as follows: D = N − F/N + F, where N is the exploration time for the novel object, F is that for the familiar object, and N + F is the total exploration time for both objects during T2 [53]. Correct recognition was shown by rats consistently spending more time inspecting a novel object than the familiar one during T2 [29].

In all the above-described experiments, to avoid the presence of olfactory cues, the apparatuses and objects (where necessary) were carefully cleaned with 20% ethanol after each trial and then wiped with dry paper.

### 4.3. Drugs

**Figure 5:**
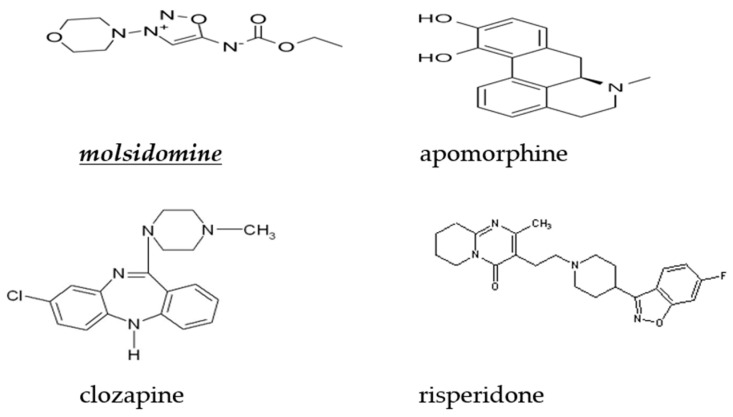
Chemical structure of the nitric oxide donor molsidomine, apomorphine, clozapine, and risperidone.

Apomorphine hydrochloride was dissolved in saline (NaCl 0.9%) that contained 0.1% ascorbic acid to prevent oxidation. Molsidomine (Sigma, St. Louis, MO, USA) was dissolved in saline (NaCl 0.9%). Clozapine (Sigma, St. Louis, MO, USA) and risperidone (Sigma, St. Louis, MO, USA) were dissolved in a minimum volume of acetic acid, made up to volume with saline, and pH adjusted to 7 with 0.1 M NaOH. All drug solutions were freshly prepared on the day of testing and were administered intraperitoneally (i.p.) in a volume of 1 mL/kg. For all studies, control rats received isovolumetric quantities of the specific vehicle solutions. The chemical structures of molsidomine, clozapine, and risperidone are illustrated in Figure 5.

### 4.4. Experimental Protocol

Daily testing was conducted between 10 a.m. and 3 p.m. during the light phase of the light/dark cycle. The animals’ behavior was video-recorded. Data evaluation was performed by a researcher who was familiar with the pharmacological treatment.

#### 4.4.1. Experiment 1: Effects of Acute Treatment with Molsidomine on Attenuating Apomorphine-Induced Performance Deficits in the OLT

Appropriate treatment was performed just after the training (sample) trial T1. Molsidomine was administered 5–10 s after the respective vehicle or apomorphine. The doses of molsidomine (2 and 4 mg/kg) were selected based on previous reports [7,19,20]. The dose of apomorphine (0.5 mg/kg) was selected based on a previous study in which it was found to impair rats’ performance in the OLT without producing side effects [30].

In this study, the 2 h ITI was selected since, at this delay condition, spatial recognition memory abilities are still intact in the vehicle-treated rat [30].

Animals were randomly divided into six experimental groups with 8 rats per group as follows: vehicle (saline) + vehicle (saline + 0.1% ascorbic acid); vehicle (saline) + molsidomine (2 mg/kg); vehicle (saline) + molsidomine (4 mg/kg); vehicle (saline + 0.1% ascorbic acid) + apomorphine (0.5 mg/kg); apomorphine (0.5 mg/kg) + molsidomine (2 mg/kg); and apomorphine (0.5 mg/kg) + molsidomine (4 mg/kg).

#### 4.4.2. Experiment 2: Effects of Acute Treatment with Molsidomine on Attenuating Apomorphine-Induced Performance Deficits in the STPAT

Appropriate treatment was performed for 40 min before starting the training trial on day 1. Molsidomine was administered 5–10 s after the respective vehicles or apomorphine. The doses of molsidomine (2 and 4 mg/kg) were selected based on previous reports [7,19,20]. The dose of apomorphine (0.5 mg/kg) was selected based on prior results in which it was found to impair rats’ performance in the STPAT without producing side effects [23,30].

Animals were randomly divided into six experimental groups with 8 rats per group as follows: vehicle (saline) + vehicle (saline + 0.1% ascorbic acid); vehicle (saline) + molsidomine (2 mg/kg); vehicle (saline) + molsidomine (4 mg/kg); vehicle (saline + 0.1% ascorbic acid) + apomorphine (0.5 mg/kg); apomorphine (0.5 mg/kg) + molsidomine (2 mg/kg); and apomorphine (0.5 mg/kg) + molsidomine (4 mg/kg).

#### 4.4.3. Experiment 3: Effects of Acute Treatment with Sub-Threshold Doses of Molsidomine and Clozapine on Attenuating Apomorphine-Induced Performance Deficits on the ORT

Appropriate treatment was performed just after the training (sample) trial T1. Molsidomine was administered 5–10 s after the respective vehicles or apomorphine. Clozapine was injected 5–10 s after molsidomine. The sub-effective doses of molsidomine (1 mg/kg) and clozapine (0.1 mg/kg) were selected on the basis of previous studies [20,55]. Apomorphine’s dose (0.5 mg/kg) was selected based on a previous report in which it was found to impair rats’ performance in the ORT without producing side effects [30].

In this study, the 2 h ITI was selected since, at this delay condition, spatial recognition memory abilities are still intact in the vehicle-treated rat [7,30].

Animals were randomly divided into four experimental groups with 8 rats per group as follows: vehicle (saline) + vehicle (saline + 0.1% ascorbic acid) + vehicle (saline + acetic acid + NaOH 0.1 M); vehicle (saline) + molsidomine (1 mg/kg) + clozapine (0.1 mg/kg); vehicle (saline) + vehicle (saline + acetic acid + NaOH 0.1 M) + apomorphine (0.5 mg/kg) and apomorphine (0.5 mg/kg) + molsidomine (1 mg/kg) + clozapine (0.1 mg/kg).

#### 4.4.4. Experiment 4: Effects of Acute Treatment with Sub-Threshold Doses of Molsidomine and Risperidone on Natural Forgetting Assessed in the ORT

Chemicals were administered immediately after sample trial T1. Risperidone was administered 5–10 s after the respective vehicles or molsidomine. The sub-effective doses of molsidomine (1 mg/kg) and risperidone (0.03 mg/kg) were selected on the basis of previous studies [20,56].

In this study, the 24 h ITI was utilized since, at this delay condition, non-spatial recognition memory abilities are extinguished in the normal rat [20].

Animals were randomly divided into four experimental groups with 8 rats per group as follows: vehicle (saline) + vehicle (saline + acetic acid + NaOH 0.1 M); vehicle (saline) + molsidomine (1 mg/kg); vehicle (saline + acetic acid + NaOH 0.1 M) + risperidone (0.03 mg/kg); and molsidomine (1 mg/kg) + risperidone (0.03 mg/kg).

#### 4.4.5. Experiment 5: Effects of Acute Treatment with Sub-Threshold Doses of Molsidomine and Risperidone on Apomorphine-Induced Non-Spatial Recognition Memory Deficits Assessed in the ORT

Appropriate treatment was performed just after the training (sample) trial T1. Molsidomine was administered 5–10 s after the respective vehicles or apomorphine. Risperidone was injected 5–10 s after molsidomine. The dose of apomorphine (0.5 mg/kg) was selected based on a previous report in which it was found to impair rats’ performance in the OLT without producing side effects [30]. The sub-effective doses of molsidomine (1 mg/kg) and risperidone (0.03 mg/kg) were selected based on previous studies [20,56].

In this study, the 2 h ITI was selected since, at this delay condition, non-spatial recognition memory abilities are still intact in the vehicle-treated rat [7,30].

Animals were randomly divided into four experimental groups with 8 rats per group as follows: vehicle (saline) + vehicle (saline + 0.1% ascorbic acid) + vehicle (saline + acetic acid + NaOH 0.1 M); vehicle (saline) + molsidomine (1 mg/kg) + risperidone (0.03 mg/kg); vehicle (saline) + vehicle (saline + acetic acid + NaOH 0.1 M) + apomorphine (0.5 mg/kg) and apomorphine (0.5 mg/kg) + molsidomine (1 mg/kg) + risperidone (0.03 mg/kg).

### 4.5. Statistical Analysis

Experiments 1, 3, 4, and 5 data were expressed as mean ± S.E.M. and were calculated using a two-way analysis of variance (ANOVA). Post-hoc comparisons between treatment means were made using the Tukey’s *t* test, but only when a significant interaction was achieved.

Experiment 2 data were expressed as medians and interquartile ranges. Pre-shock and retention latencies were evaluated using the Kruskal–Wallis non-parametric test. Post hoc comparisons were made by Dunn’s test. Values of *p* < 0.05 were considered statistically significant [57].

## 5. Conclusions

The findings here presented suggest that the NO donor molsidomine counteracts cognitive impairments associated with DAergic abnormalities. These results might have translational usefulness since molsidomine’s effects were evidenced in preclinical behavioral paradigms mimicking the cognitive deficits and distinctive features of schizophrenia patients. Additionally, the effectiveness of molsidomine to potentiate the anti-schizophrenia-like action of the atypical antipsychotics clozapine and risperidone has also been evidenced. The last-mentioned proposal proposes that the concomitant administration of molsidomine with a second-generation neuroleptic might constitute a novel strategy for the therapy of schizophrenia. Further research is mandatory to investigate the potential antipsychotic-like profile of molsidomine and prove a role for this compound in the therapy of this psychiatric disorder.

## Figures and Tables

**Table 1:** Pre-shock and retention latencies expressed by different groups of rats in the step-through passive avoidance test.

Treatment	Pre-Shock Latencies (s.)	Retention Latencies (s.)
Vehicle+vehicle	24 (13.75–58.5)	180 (180–180)
Vehicle+molsidomine (2 mg/kg)	30.5 (15.75–47.25)	180 (180–180)
Vehicle+molsidomine (4 mg/kg)	31.5 (12.25–62.25)	180 (180–180)
Apomorphine (0.5 mg/kg) + vehicle	30 (25.75–45.75)	40.5 (15.5–70) *
Apomorphine+molsidomine (2 mg/kg)	32 (13.25–47)	93 (67.75–180)
Apomorphine+molsidomine (4 mg/kg)	30 (22.25–52.25)	180 (34.5–180)

Data are expressed as medians and interquartile ranges of 8 rats per treatment group. * *p* < 0.05 vs. all the other groups.

## Data Availability

The data presented in this study are available on request from the corresponding author.

## Sample Availability

Samples of apomorphine, clozapine, molsidomine, and risperidone are available from the authors.
